# In vitro Cytotoxic and Antimicrobial Activity of Essential Oil From Satureja Intermedia

**DOI:** 10.5812/ircmj.4989

**Published:** 2013-01-05

**Authors:** Iman Sadeghi, Morteza Yousefzadi, Mehrdad Behmanesh, Mozafar Sharifi, Aiuob Moradi

**Affiliations:** 1 Department of Genetics, Faculty of Biological Sciences, Tarbiat Modares University, Tehran, IR Iran; 2 Department of Biology, Faculty of Basic Sciences, Hormozgan University, Bandar Abbas, IR Iran; 3 Department of Plant Biology, Faculty of Biological Sciences, Tarbiat Modares University, Tehran, IR Iran; 4 Agricultural and Natural Resources Research Center, Gilan, IR Iran

**Keywords:** Satureja, Gas Chromatography-Mass Spectrometry, Thymol

## Abstract

**Background:**

Many members of the genus *Satureja* have aromatic and medicinal characteristics. Objectives

**Objectives:**

The purpose of the present work was to determine cytotoxic activity of the essential oil of *S. intermedia* CA Mey (Lamiaceae) on two human cancerous cell lines and its in vitro inhibitory effects against 11 pathogenic bacteria and fungi as well.

**Materials and Methods:**

The essential oil was isolated by hydrodistillation and analyzed by combination of capillary GC-FID and GC-MS. The in vitro toxicological study was based on the MTT cytotoxicity assay and antimicrobial activity of the essential oil was studied according to the disc diffusion method and MIC value.

**Results:**

Thymol (34.5%), γ-terpinene (18.2%) and ρ-cymene (10.5%) were the main components of the essential oil. The toxicological study on 5637 and KYSE cell lines showed IC50 values of 156 μg/ml. The essential oil exhibited considerable antimicrobial activity on tested bacteria and fungi.

**Conclusions:**

From the results of the present study, it may be concluded that the essential oil of *S. intermedia* and its major constitutes are interesting in antibacterial and anticancer applications.

## 1. Background

Essential oils are complex mixtures of odorous and volatile compounds obtained from many plants and have recently gained popularity and scientific interest. Aromatic plants have long been used for spices and natural food conservancies, in the perfume industry and aromatherapy and for different medical purposes. Researchers have been interested in biologically active compounds isolated from plant species for the elimination of pathogenic microorganisms because of the resistance that microorganisms have built against current antibiotics. Also some essential oils were shown to elicit cytotoxicity and induce apoptosis on the cancerous cells. Based on cytotoxicity bioassay, over 400 compounds have been isolated from plants (1, 2).

The genus *Satureja* L. (Lamiaceae) comprises more than 200 species of aromatic herbs and shrubs, mainly distributed in the Mediterranean region. This genus is represented by 14 species from which 8 species are endemic and are distributed commonly in mountainous regions of Iran (3-5). Many members of the genus *Satureja* have aromatic and medicinal properties. The essential oil compositions and antimicrobial activities of some *Satureja* species have been studied (6-9). These studies have revealed that the *Satureja* species have antimicrobial activity against human, food, and plant pathogens due to the presence of phenolic components such as thymol and carvacrol (10, 11).

Earlier studies revealed that the essential oil of *Satureja* species are rich in carvacrol, γ-terpinene, thymol, and p-cymene (12, 13). However the percentage or presence of some main components in the essential oils of this genus shows a noticeable diversity (14, 15). Although antimicrobial activity of the essential oil of some *Satureja* species has been previously reported, (6, 7, 10, 16) antimicrobial and cytotoxic activity of *S. intermedia* essential oil have not been studied so far.

## 2. Objectives

In this study, we reported the results of cytotoxic activity of the essential oil of *S. intermedia* and evaluation of its in vitro inhibitory effects against 8 pathogenic Gram-positive and Gram-negative bacteria in addition to three pathogenic fungi as well.

## 3. Materials and Methods

### 3.1. Plant Material

The aerial parts of S. intermedia were collected, on June 2009 at full flowering stage from Talesh, Iran, at an altitude of 1750 m. The plant material was identified by Dr. Hadian and a voucher specimen (HAPH-88121) is deposited at the Herbarium of Biology Department, Hormozgan University, Bandar Abbas, Hormozgan Province, Iran.

### 3.2. Essential Oil Isolation

The powdered plant aerial parts (250 g) were hydrodistilled using a Clevenger type apparatus for 3 hours according to the method recommended in British Pharmacopoeia. The resulting essential oil was dried over anhydrous sodium sulfate and stored at 4ºC until analyzed and tested. 

### 3.3. Essential Oil Analysis and Identification Procedure

GC-FID analyses of the oil were conducted using a Thermoquest-Finnigan instrument equipped with a DB-5 fused silica column (60 m × 0.25 mm i.d., film thickness 0.25 µm). Nitrogen was used as the carrier gas at the constant flow of 1.1 ml/min. The split ratio was 1/50. The oven temperature was raised from 60ºC to 250ºC at a rate of 5 ºC/min. The injector and detector (FID) temperatures were kept at 250ºC and 280ºC, respectively. GC-MS analysis was carried out on a Thermoquest-Finnigan Trace GC-MS instrument equipped with the same column and temperature programming as mentioned for GC. Transfer line temperature was 250ºC. Helium was used as the carrier gas at a flow rate of 1.1 ml/min with a split ratio equal to 1/50.

The constituents of the essential oils were identified by calculation of their retention indices under temperature-programmed conditions for n-alkanes (C6–C24) and the oil on a DB-5 column under the same conditions. Individual compounds were identified by comparison of their mass spectra with those of the internal reference mass spectra library (Wiley 7.0) or with authentic compounds and confirmed by comparison of their retention indices with authentic compounds or with those of reported in the literature (17). Semi-quantitative data was obtained from FID area percentages without the use of correction factors.

### 3.4. Microbial Strains

Eleven microbial strains were used in the antimicrobial activity assay, which included; *Bacillus subtilis* (ATCC 465), B. pumulis (PTCC 1274), *Enterococcus faecalis* (ATCC 29737), *Staphylococcus aureus* (ATCC 25923), *Staphylococcus epidermidis* (ATCC 12228), *Escherichia coli* (ATCC 25922), *Klebsiella pneumoniae* (ATCC 10031), *Pseudomonas aeruginosa* (ATCC 85327), *Aspergillus*

*niger* (ATCC 16404), *Candida albicans* (ATCC 10231) and *Saccharomyces cerevisiae* (ATCC 9763).

### 3.5. Antimicrobial Screening

The antimicrobial activity of the essential oil and its main component was determined by the disk diffusion method (18). Briefly, 0.1 ml of a suspension of the test microorganism (10^8^ cells/ml) was spread on Mueller-Hinton Agar plates for bacteria and Sabouraud Dextrose Agar for the fungi. Sterile 6 mm disks, containing 10µl of essential oil were placed on the microbial lawns. The plates were incubated at 37ºC for 24 hours for bacteria and 30ºC for 48 hours for fungi. The diameters of the zones of inhibition were measured and reported in mm. Triplicate tests were carried out in all experiments.

### 3.6. Determination of Minimum Inhibitory Concentration (MIC)

MIC values were determined by broth microdilution assay recommended by the NCCLS (19). Serial two-fold dilutions of the essential oil were made in Mueller-Hinton Broth containing 0.5% Tween 80 for bacteria and Sabouraud Dextrose Broth with 0.5% Tween 80 for fungi in 96-well micro titer plates. Fresh microbial suspensions prepared from overnight grown cultures in the same media were added to give a final concentration of 5 × 105 organisms/ml. Controls of medium with microorganisms or the essential oil were included. The microplates were incubated at 37ºC for 24 hours for bacteria and 30ºC for 48 hours for fungi. The first dilution with no microbial growth was recorded as MIC.

### 3.8. Cell line and Culture

The human oesophagus squamous cell carcinoma (KYSE30) and human bladder carcinoma cell line (5637) were obtained from Pasteur Institute of Iran. Cells were cultured in RPMI-1640 supplemented with 10% fetal bovine serum (Gibco) and 1% penicillin-streptomycin, at 37ºC, in humidified air containing 5% CO2.

### 3.9. Cytotoxicity Assay

Cytotoxicity was assessed by the tetrazolium-based colorimetric assay (MTT) which measures the reduction of the tetrazolium salt MTT (3-[4,5-dimethylthiazol-2-yl]-2, 5-diphenyltetrazolium bromide; Roche) into a blue formazan product, mainly by the activity of the mitochondrial enzymes, cytochrome oxidase and succinate dehydrogenase. Typically, 100 µl of cells suspension were plated at a density of approximate 2 ×10^4^ cells per well in a 96-well plate, and were subsequently incubated at 37ºC in a 5% CO2 humid incubator for 24 hours. Then essential oils with different concentrations were added to each group (triplicate wells) and the incubation was continued for 24h, followed by adding l0 µl (5mg/ml) of MTT dye solution to each well for 4 hours at 37ºC. After removal of the MTT dye solution, cells were treated with 100 µl DMSO and the absorbance at 490nm was quantified using ELISA reader. The cytotoxicity was calculated after comparing with the control (treated with 0.1% DMSO). Cytotoxicity is expressed as the concentration of drug inhibiting cell growth by 50% (IC50). All tests and analysis were run in triplicate and mean values recorded.

## 4. Results

### 4.1. Chemical Composition of Essential Oil

The essential oil was obtained by hydrodistillation from the aerial part of *S. intermedia* and then subjected to GC and GC/MS to identify its composition. Qualitative and quantitative analytical results are shown in Table 1 in which compounds are listed in order of their elution on the DB-5 column. The yield of essential oil was 0.52% (w/w) on dry weight basis. In our study, thymol (34.5%), γ-terpinene (18.2%), ρ-cymene (10.5%), limonene (7.3%), α-terpinene (7.1%), carvacrol (6.9%) and elemicine (5.3%) were the main compounds. In another research on *S. intermedia*, thymol (32.3%), γ-terpinene (29.3%) reported as the main compounds.15. The contents of carvacrol, p-cymene and specially thymol are usually present as major compounds in the oil of other *Satureja* species (20-22). These differences may be due to several reasons: genetic diversity, climatic conditions, ecological differences (23).

**Table 1:** Main Components of the Essential Oil of S. intermedi

**Compound**	**Retention index**	** Percentage**
** Thymol**	1290	34.5
** γ-Terpinene**	1062	18.2
** Ρ-Cymene**	1024	10.5
** Limonene**	1031	7.3
** α-Terpinene**	1018	7.1
** Carvacrol**	1297	6.9
** Elemicine**	1554	5.3

### 4.2. Antimicrobial Activity

Inhibition zones (IZ) and minimum inhibitory concentration (MIC) values of the essential oil, its major compound and standard antibiotic are shown in Table 2. The results of the antimicrobial activities of the essential oil indicated that the oil exhibited moderate to high antimicrobial activity. According to these results, essential oil of this species exhibited considerable antimicrobial activity on tested bacteria and fungi except for two resistant microbial strains: A. *niger* (fungus) and P. aeruginosa (Gram-negative bacteria). The data showed that B. pumulis, B. subtilis and S. epidermidis were the most sensitive microorganisms to the oil with the inhibition zones 36, 35 and 33 mm and MIC values of 0.465, 0.465 and 0.93 mg/ml, respectively. Among bacteria P. aeruginosa, E. faecalis and K. pneumonia were the most resistant bacteria against the essential oil.

**Table 2:** Antimicrobial Activity of Essential Oils From S. intermedia

**Microorganisms a**	***S. intermedia***		**Thymol**		**Standard antibiotics**	
		**IZ b**	**MIC c**	**IZ**	**MIC**	**Ampicillin d**	**Nystatine e**
*B. subtilis*	35 ± 0.3	0.465	46 ± 0.5	0.23	14 ± 0.4	-
*B. pumulis*	36 ± 0.2	0.465	47 ± 0.3	0.23	15 ± 0.3	-
*E. faecalis*	12 ± 0.2	7.5	25 ± 0.2	1.75	11 ± 0.3	-
*S. aureus*	20 ± 0.3	3.75	34 ± 0.2	0.465	13 ± 0.3	-
*S. epidermidis*	33 ± 0.5	0.93	38 ± 0.4	0.465	19 ± 0.5	-
*E. coli*	12 ± 0.3	7.5	32 ± 0.5	0.465	12 ± 0.2	-
*K. pneumoniae*	11 ± 0.4	7.5	24 ± 0.6	1.75	-	-
*P. aeruginosa*	0	-	12 ± 0.2	7.5	10 ± 0.3	-
*A. niger*	0	-	10	7.5	-	16 ± 0.4
*C. albicans*	13 ± 0.5	7.5	14	3.75	-	18 ± 0.5
*S. cerevisiae*	15 ± 0.7	3.75	18	1.75	-	18 ± 0.2

^a^Inactive (-); moderately active (7 - 14); highly active (> 14); nt, not tested. Values are given as mean ± standard deviation

^b^Inhibition Zone includes diameter of disc (6 mm)

^c^Minimum inhibitory concentration values as mg/ml

^d^Tested at 10 μg/disc

^e^Tested at 30 μg/disc

Analysis of antimicrobial activity of the essential oil on fungi also revealed that A. niger is absolutely insensitive to essential oil however S. cervisiae showed to be the most sensitive fungus against essential oil with IZ and MIC values as 15 mm and 3.75 mg/ml, respectively. Although the essential oil of the present species showed both antifungal and antibacterial activity, but the IZ and MIC values for fungi were much higher than that of bacteria which shows the higher antibacterial potent of the essential oil comparing to antifungal activity. By comparing the antimicrobial activity of the essential oil of *S. intermedia* in this study with its composition, it can be suggested that thymol which is the major components of the essential oil of this plant might be responsible compounds for antimicrobial activity of this species. Antimicrobial activity of thymol is considered as a major component of the oil tested. The result showed that thymol exhibit antibacterial activity is stronger than antifungal activity (Table 2).

## 5. Discussion

Antimicrobial activity of other *Satureja* species on our tested bacteria or fungi or other types of microorganisms have been previously studied. Sonboli et al. (22), reported that the antimicrobial activity of essential oil of S. laxiflora which thymol was the main component of the essential oil proposed to be responsible for its antimicrobial activity. The effect of S. montana and S. *subspicata* oils were also reported and essential oils extracted from S. spicigera, S. biflora, S. masukensis and S. pseudosimensis showed a high inhibition against a wide range of microorganisms (7, 16, 24). It has been proposed that the essential oils which are rich in phenolic compounds may exert a high antimicrobial activity (25).

**Figure 1:**
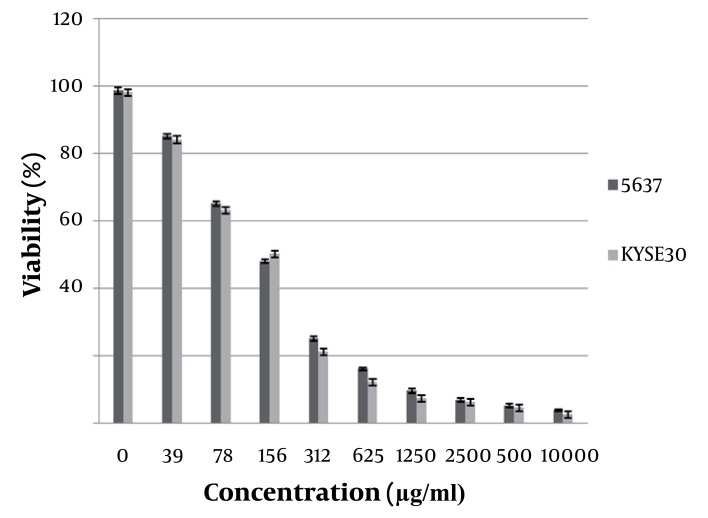
Cytotoxicity of the Essential Oil From *S.intermedia*. Viability Values Were Expressed as the Mean ± S.D., Determined From the Results of MTT Assay in Triplicate Experiments

To evaluate the effect of essential oils on cell viability, cytotoxicity assay was performed, increasing drug concentrations and the cell viability were determined by the MTT assay. For these assays, cultured cells were exposed to essential oil for 24hours. The results of cytotoxicity of the essential oil of *S. intermedia* on the human bladder carcinoma cell line (5637) and human oesopphagus squamous cell carcinoma (KYSE30) cell lines after 24hours exposure at dosages ranging from 39 to 1000 μg/ml were shown in Figure 1. Essential oil significantly reduced cell viability of KYSE30 and 5637 cells in a dose-dependent manner starting 39 to 1000 μg/ml with IC50 156 μg/ml.

Cytotoxicity of thyme essential oil was investigated on the HNSCC and UMSCC cell lines. Essential oils and their individual volatile components have been brought the attention of research groups on cancer (26). A number of articles are devoted to investigate their effect against a variety of human cancer cell lines. Numerous reports showed the high cytotoxic properties of terpenes, and phenolics against cancer cell lines (27). The IC50 of thyme essential oil extract was 369 μg/ml (28). In another study, the genotoxic effects of thymol were investigated in human peripheral lymphocytes treated with 25, 50, 75, and 100 μg/ml concentrations. Thymol induced structural chromosome aberration and frequency of micronucleus at all concentrations (29).

Our result in this study represented that the essential oil of *S. intermedia* like other mentioned *Satureja* species in this paper has a great potent for antimicrobial properties in addition to cytotoxic activities. These data indicate the possibility that *S. intermedia* oil and its constituents may be applied as an antibacterial agent and moreover an anticancer agent.
